# Mutagenesis and homologous recombination in *Drosophila* cell lines using CRISPR/Cas9

**DOI:** 10.1242/bio.20137120

**Published:** 2013-12-06

**Authors:** Andrew R. Bassett, Charlotte Tibbit, Chris P. Ponting, Ji-Long Liu

**Affiliations:** Medical Research Council Functional Genomics Unit, Department of Physiology, Anatomy and Genetics, University of Oxford, South Parks Road, Oxford OX1 3PT, UK

**Keywords:** Genome engineering, CRISPR, Cas9, *Drosophila* S2 cells, Homologous recombination, Gene targeting

## Abstract

We have applied the CRISPR/Cas9 system to *Drosophila* S2 cells to generate targeted genetic mutations in more than 85% of alleles. By targeting a constitutive exon of the *AGO1* gene, we demonstrate homozygous mutation in up to 82% of cells, thereby allowing the study of genetic knockouts in a *Drosophila* cell line for the first time. We have shown that homologous gene targeting is possible at 1–4% efficiency using this system, allowing for the construction of defined insertions and deletions. We demonstrate that a 1 kb homology arm length is optimal for integration by homologous gene targeting, and demonstrate its efficacy by tagging the endogenous AGO1 protein. This technology enables controlled genetic manipulation in *Drosophila* cell lines, and its simplicity offers the opportunity to study cellular phenotypes genome-wide.

## Introduction

Genome engineering technologies permit precise alterations of eukaryotic genomes thereby enabling more directed and more nuanced studies of gene function. The ability to perform such manipulations in essentially any organism has been driven by the use of nucleases that can be targeted to specific sites within the genome in a predictable manner (reviewed by Gaj et al., 2013). These can create double strand breaks (DSB) leading to enhanced rates of DNA repair at the targeted site by either non-homologous end joining (NHEJ) or homologous recombination (HR) (Bibikova et al., 2002). Both mechanisms can be exploited to study gene function. The NHEJ repair mechanism occasionally results in the insertion or deletion of a few bases at the DSB site, which can shift reading frame in proteins or remove functionality from transcription factor binding or splice sites. If supplied with exogenous DNA repair templates, HR can be used to engineer directed changes at defined loci (Beumer et al., 2006; Beumer et al., 2008; Bibikova et al., 2003). The ability to design site-specific nucleases allows the generation of targeted mutations and homologous integrations in systems that have hitherto remained refractory to such manipulations.

**Fig. 1.**
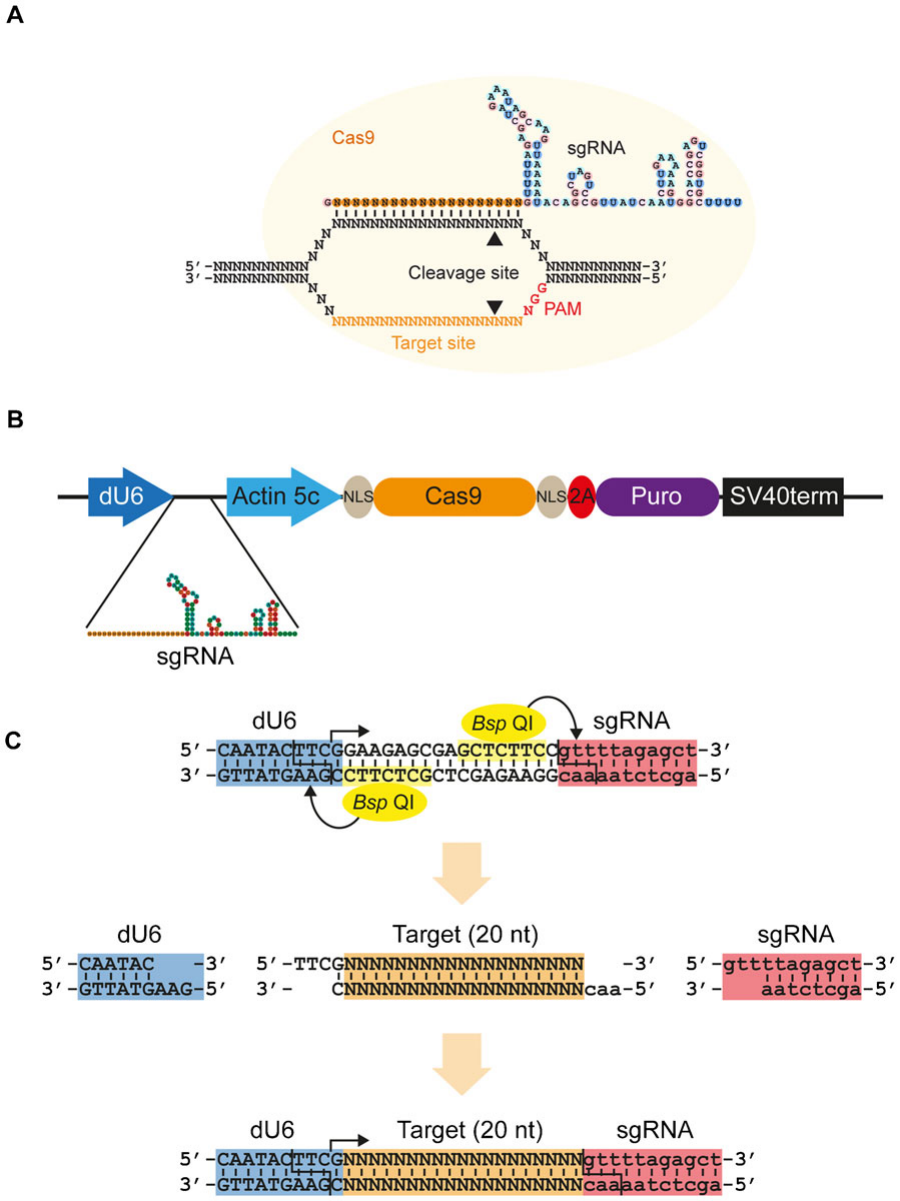
CRISPR/Cas9 expression system for *Drosophila* cell culture. (A) The CRISPR/Cas9 system adapted from *S. pyogenes* for inducing double strand breaks. The synthetic guide RNA (sgRNA) contains 20 nt complementarity to the target site within the DNA, and the RNA structure necessary for incorporation into the Cas9 protein. Cas9 is indicated by a yellow circle, cleavage sites by arrowheads and protospacer adjacent motif (PAM, NGG) required for cleavage in red. (B) Schematic of the expression vector. The sgRNA is produced from a *Drosophila* U6 promoter by RNA polymerase III, which produces an uncapped transcript. Human codon-optimised Cas9 mRNA (orange oval) containing N- and C-terminal SV40 nuclear localisation signals (grey ovals) is produced from the strong, constitutive actin5C promoter by RNA polymerase II as the first half of a bicistronic transcript with the puromycin N-acetyltransferase gene (purple oval). The two open reading frames are separated by a viral 2A ribosome skipping site (red oval) to allow bicistronic expression, and transcription is terminated by a polyadenylation signal from the SV40 virus. (C) Strategy for cloning of target oligos. Two *Bsp* QI sites (yellow) cause cleavage at the end of the U6 promoter (blue) and sgRNA backbone (red), leaving 3 nt 5′ overhangs. Transcription from the dU6 promoter begins with the indicated G nucleotide (arrow). Target oligos (orange) are designed to provide complementary overhangs flanking the 20 nt target sequence. If the target sequence does not begin with a G, this is appended to its 5′ end to reconstitute the G nucleotide required by the dU6 promoter (as indicated here). See also supplementary material Table S1.

The type II CRISPR/Cas9 (clustered regularly interspaced short palindromic repeats/CRISPR-associated) system of viral defence in bacteria (Barrangou et al., 2007; Ishino et al., 1987) has recently been adapted for genome engineering in many organisms including zebrafish (Hwang et al., 2013; Xiao et al., 2013), mouse (Wang et al., 2013; Yang et al., 2013) and *Drosophila* (Bassett et al., 2013; Gratz et al., 2013; Kondo and Ueda, 2013; Ren et al., 2013; Sebo et al., 2013; Yu et al., 2013). The Cas9 endonuclease from *Streptococcus pyogenes* can be targeted using a short synthetic guide RNA (sgRNA) to generate a double strand break at a specific site in the genome (Cong et al., 2013; Jinek et al., 2012; Mali et al., 2013b). This sgRNA contains a 20 nucleotide target sequence that determines specificity through complementary base pairing with the DNA. The Cas9 protein additionally requires a protospacer adjacent motif (PAM) of NGG to occur within the DNA adjacent to the target sequence to achieve efficient endonucleolytic cleavage (Fig. 1A). This system has only a short specificity determinant and relatively relaxed targeting rules (Cradick et al., 2013; Fu et al., 2013), making its application to large genomes more difficult due to off target mutagenesis (Cho et al., 2013; Hsu et al., 2013; Ran et al., 2013). This has prompted development of the “double nick” technique that requires the coordinated activity of a pair of mutated Cas9 proteins targeted to neighbouring sequences, to improve sequence specificity (Mali et al., 2013a; Ran et al., 2013). Whilst this could be applied to smaller genomes such as *Drosophila*, it is possible that even a single sgRNA may be sufficient so long as sgRNA sequences are carefully chosen to minimise off target effects (Bassett et al., 2013; Gratz et al., 2013; Ren et al., 2013), although more rigorous studies will be necessary to establish the extent and nature of off targeting in *Drosophila* cells.

The CRISPR/Cas9 system has recently been adapted by us and others to engineer short and long deletions in chosen *Drosophila* genes (Bassett et al., 2013; Gratz et al., 2013; Kondo and Ueda, 2013; Ren et al., 2013; Sebo et al., 2013; Yu et al., 2013). Here we describe its application to *Drosophila* cell culture. This now allows analysis of cellular phenotypes resulting from the targeted mutation of a gene that may be difficult or impossible to perform in the context of a whole organism. This may be due to the complexities of dealing with a mixture of multiple cell types, or embryonic lethality of certain mutations preventing analysis at an appropriate stage. The simplicity, speed and efficiency of the generation of genetic mutations also allow screening for candidate genes involved in particular cellular processes. This system provides a powerful alternative to currently available RNAi screens, which are limited to providing only partial knockdown of function at a post-transcriptional stage.

Furthermore, our demonstration of homologous gene targeting permits the precise manipulation of endogenous cellular genomes. This has many applications in gene deletion, analysis of defined genetic changes, visualisation, purification and expression analysis of genes or their protein products in an endogenous cellular context that retains all of the regulatory elements present within the genome.

## Results

### A gene deletion system for *Drosophila* cells

We designed an expression vector similar to those previously used in mammalian cells (Cong et al., 2013) but using the *Drosophila* U6 promoter (Wakiyama et al., 2005) to drive pol III dependent transcription of a chimeric synthetic guide RNA (sgRNA) and the constitutive actin 5c promoter to drive Cas9 mRNA expression (Fig. 1B). Due to the relatively low efficiency of transfection of *Drosophila* cell lines we also included a puromycin N-acetyltransferase gene downstream of the Cas9 gene, separated from it by a viral derived 2A ribosome skipping site (González et al., 2011). This enables selection for Cas9 expression with puromycin, since the two proteins are produced as part of the same bicistronic transcript derived from the single actin 5c promoter.

Two *Bsp* QI restriction enzyme sites were introduced within the sgRNA to enable cloning of a pair of annealed 23–24 nt oligonucleotides that generate the target sequence of the sgRNA. Since this enzyme cuts outside of its recognition site, it removes the recognition site during cloning and thus avoids the insertion of any additional sequence. Oligonucleotides are designed by taking the 20 nt target sequence upstream of the PAM (NGG), and adding complementary overhangs to enable cloning into the *Bsp* QI site (Fig. 1C). The U6 promoter requires the first base of the transcript to be a guanine, so this either constitutes the first base of the target sequence, or can be appended to it. Consistent with the results of others, we have observed that this only marginally affects cleavage efficiency by the sgRNA (Cong et al., 2013), and greatly increases the choice of target sites within the genome, which are therefore restricted only by the requirement of the NGG protospacer adjacent motif (PAM) sequence (Fig. 1A).

### Highly efficient mutagenesis at the *yellow* locus

**Fig. 2.**
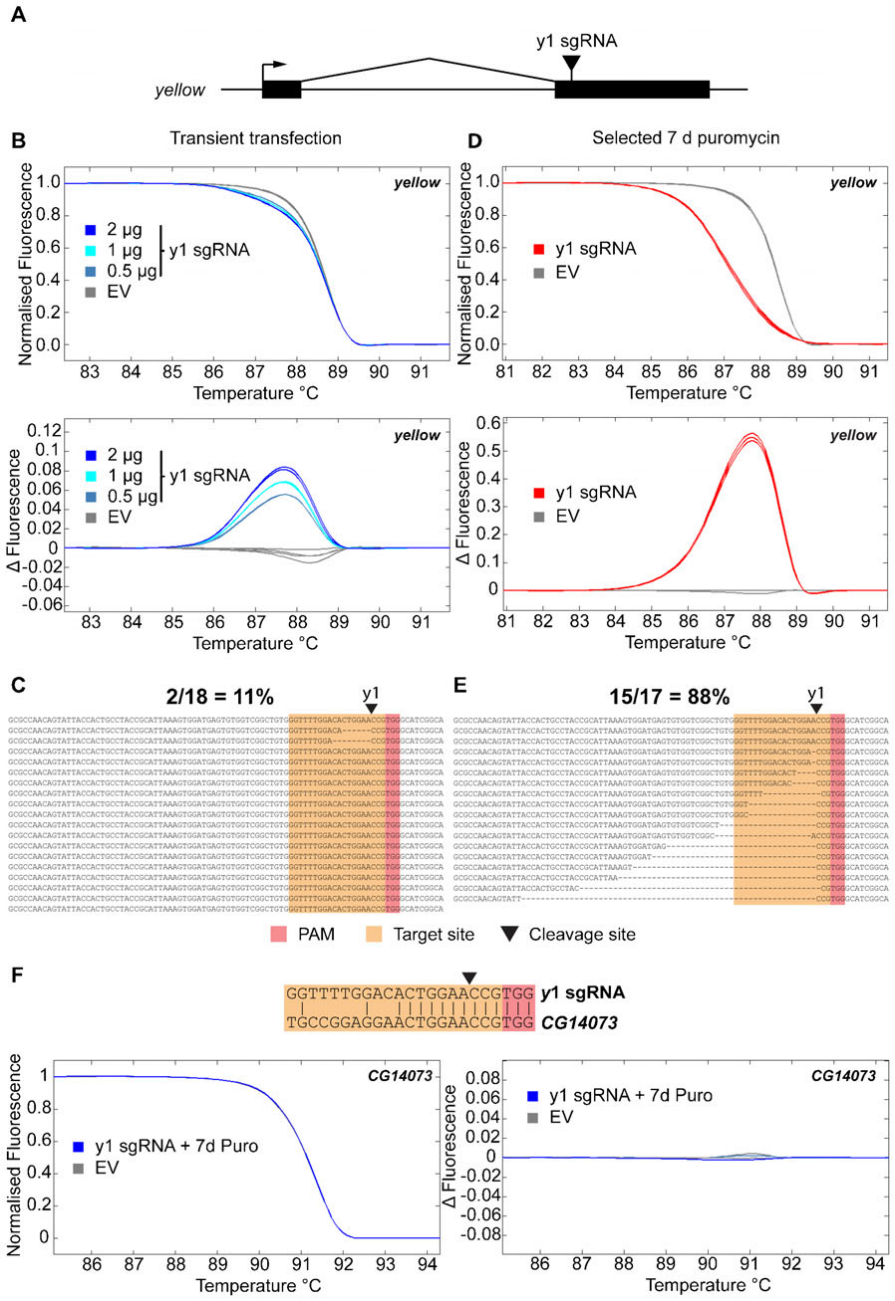
Mutagenesis of the *yellow* gene is highly efficient. (A) Schematic of the *yellow* gene showing position of sgRNA target site. Exons are indicated as black boxes, transcriptional start site by an arrow and the *y*1 sgRNA target site by a black triangle. (B) Indel detection by high resolution melt analysis (HRMA) after transient transfection. Cells were transfected with empty vector lacking sgRNA (EV, grey lines) or with different amounts of vector expressing the *y*1 sgRNA (2, 1, 0.5 µg, blue lines). DNA was analysed by HRMA 3 days post-transfection, and indicated as melting curves (upper panel) or change relative to control (lower panel). (C) Sequencing of indel mutations after transient transfection. PCR products spanning the cleavage site (black triangle) were cloned and sequenced from cells transfected with 2 µg *y*1 sgRNA vector, and showed deletions in 2 of 18 clones sequenced at the expected site. Target site is highlighted in orange, and PAM in red. The first line of each alignment indicates the wt sequence. (D) As panel B for cells selected for a further 7 days in puromycin. (E) As panel C for cells selected for a further 7 days in puromycin. (F) Lack of off target mutagenesis at the *CG14073* gene. Base pairing interactions between the *y*1 sgRNA and the site in *CG14073* are indicated (target site indicated in orange, PAM in red, cleavage site as a black triangle). HRMA analysis at the closest off-target site in the *Drosophila* genome indicates a lack of detectable indel mutants even after 7 days selection in puromycin (as panels D,E). Primer sequences are indicated in supplementary material Table S1.

We tested our system by using a sgRNA targeted against the *yellow* locus, which is known to exhibit high cleavage efficiency in flies (Fig. 2A) (Bassett et al., 2013), and detected the insertion and deletion (indel) mutations generated through imperfect non-homologous end joining (NHEJ) by high resolution melt analysis (HRMA, Fig. 2B,D). As increasing amounts of expression vector were transfected, the numbers of generated indel mutations increased (Fig. 2B). Sequencing of PCR products spanning the cleavage site showed that transient expression generated mutations in approximately 11% of alleles at 3 d post transfection (Fig. 2C). Upon selection of these cells for a further 7 d in puromycin to remove untransfected cells and to force integration of the vector into the genome, we observed a large increase in the efficiency of mutagenesis (Fig. 2D). Sequencing across the cleavage site demonstrated mutational events in 88% of alleles (Fig. 2E). Mutagenesis was not observed when empty vector lacking the sgRNA targeting sequence was used (Fig. 2B,D). Recent observations of off target cleavage by the CRISPR/Cas9 system (Cho et al., 2013; Cradick et al., 2013; Fu et al., 2013; Hsu et al., 2013; Ran et al., 2013) prompted us to test for mutagenesis at the site with the closest match to the *y*1 sgRNA (*CG14073*). We also observed no detectable off-target mutagenesis by HRMA even after 7 d selection in puromycin (Fig. 2F), although we cannot exclude the possibility that this may happen at other sites.

Consequently, puromycin selection for different lengths of time can be used to regulate the proportion of mutant alleles within the population of cells. We note that selection may not be desirable in all instances, because it will result in integration of the vector into the genome. Nevertheless, once indels have been generated by this technique, the target site will be removed and the sgRNA will not be able to target Cas9 to this site, so the integrated vector should be unable to cause further mutational events.

### Homologous integration at the *yellow* locus

**Fig. 3.**
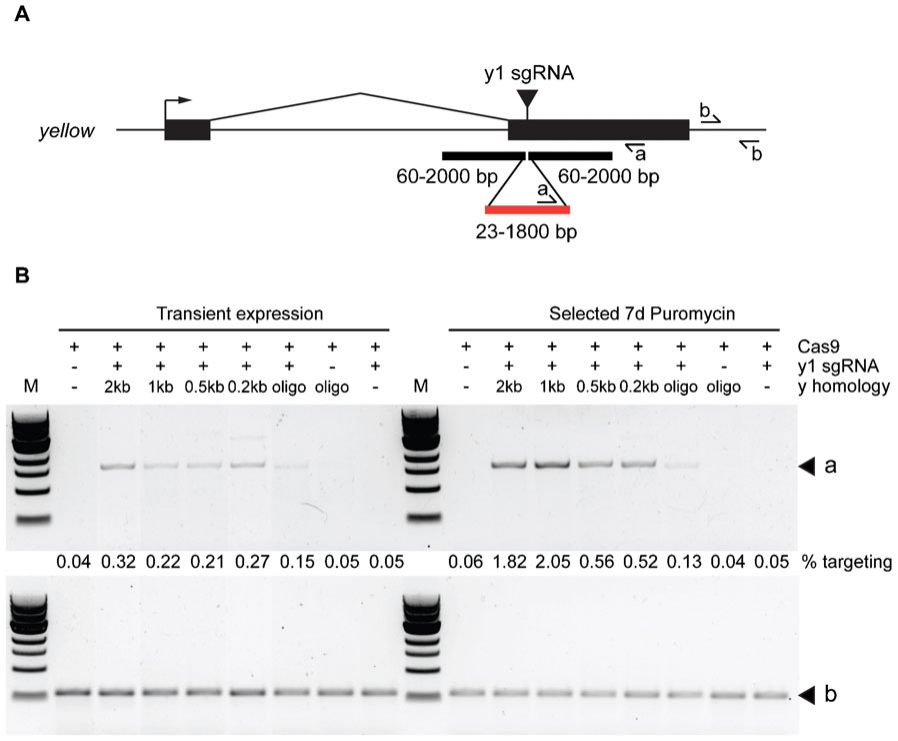
Homologous recombination at the *yellow* gene. (A) Schematic diagram of the *yellow* gene. Exons are indicated by black boxes, *y*1 sgRNA target site as a black triangle, and position of homology arms by black boxes. Inserted sequence is indicated by a red box. PCR primer pairs used in Fig. 3B are indicated by arrows and a or b. Primer sequences are indicated in supplementary material Table S1 (aF, aR, bF, bR). (B) Integration of homology constructs at target site as detected by PCR. PCR was performed after transfection of cells with the indicated constructs to detect integration (upper panel, a) or as a control, outside of the integration site (lower panel, b). Cells were harvested 3 days after transfection (left, transient expression) or after a further 7 days selection in puromycin (right, selected 7 d puromycin). Integration was observed with all homology constructs, but only upon cotransfection with plasmid expressing both Cas9 and the *y*1 sgRNA. Efficiencies of correct integration are indicated (% targeting), and were normalised to control PCR product (lower panel, b). Primer sequences are indicated in supplementary material Table S1.

We next investigated whether homologous integration is possible in S2 cells, as has been demonstrated for human and mouse cell lines (Chen et al., 2011; Cong et al., 2013; Wang et al., 2013). Previous studies have suggested that both short single stranded (ss) DNA oligonucleotides and longer double stranded (ds) DNA donors can be used as templates for homologous recombination in *Drosophila* and other organisms (Bedell et al., 2012; Beumer et al., 2013; Chen et al., 2011; Cong et al., 2013; Wang et al., 2013). We therefore cotransfected DNA donors with homology either side of the *y*1 sgRNA cleavage site with the *y*1 sgRNA/Cas9 expression vector, and assessed integration efficiency by PCR (Fig. 3). We used dsDNA donor plasmids with approximately 200, 500, 1000 or 2000 bp homology on either side of the cut site to insert a 1.8 kb DNA fragment, or a short 100 nt ssDNA oligonucleotide to insert a 23 nt sequence into the genome (Fig. 3A). Transient expression gave low but detectable integration efficiencies of around 0.3% for the longest homology arm, but after selection in puromycin to enrich for transfected cells this was increased to around 2% of alleles. As homology arm length was decreased, integration efficiency also decreased, and the oligonucleotides were integrated at a low efficiency of approximately 0.15% (Fig. 3B). The optimal length for integration efficiency and ease of detection was a dsDNA donor with 1 kb homology either side of the inserted sequence, consistent with previous results when the DSB was induced with a zinc finger nuclease (Beumer et al., 2013). Since it is possible that DNA sequences may be incorporated by direct ligation at the site of the break, we sequenced PCR products spanning the homology arms. This showed that correct homologous integration had occurred in all cases, including with the short ssDNA oligonucleotides.

### Mutations of the *AGO1* gene

**Fig. 4.**
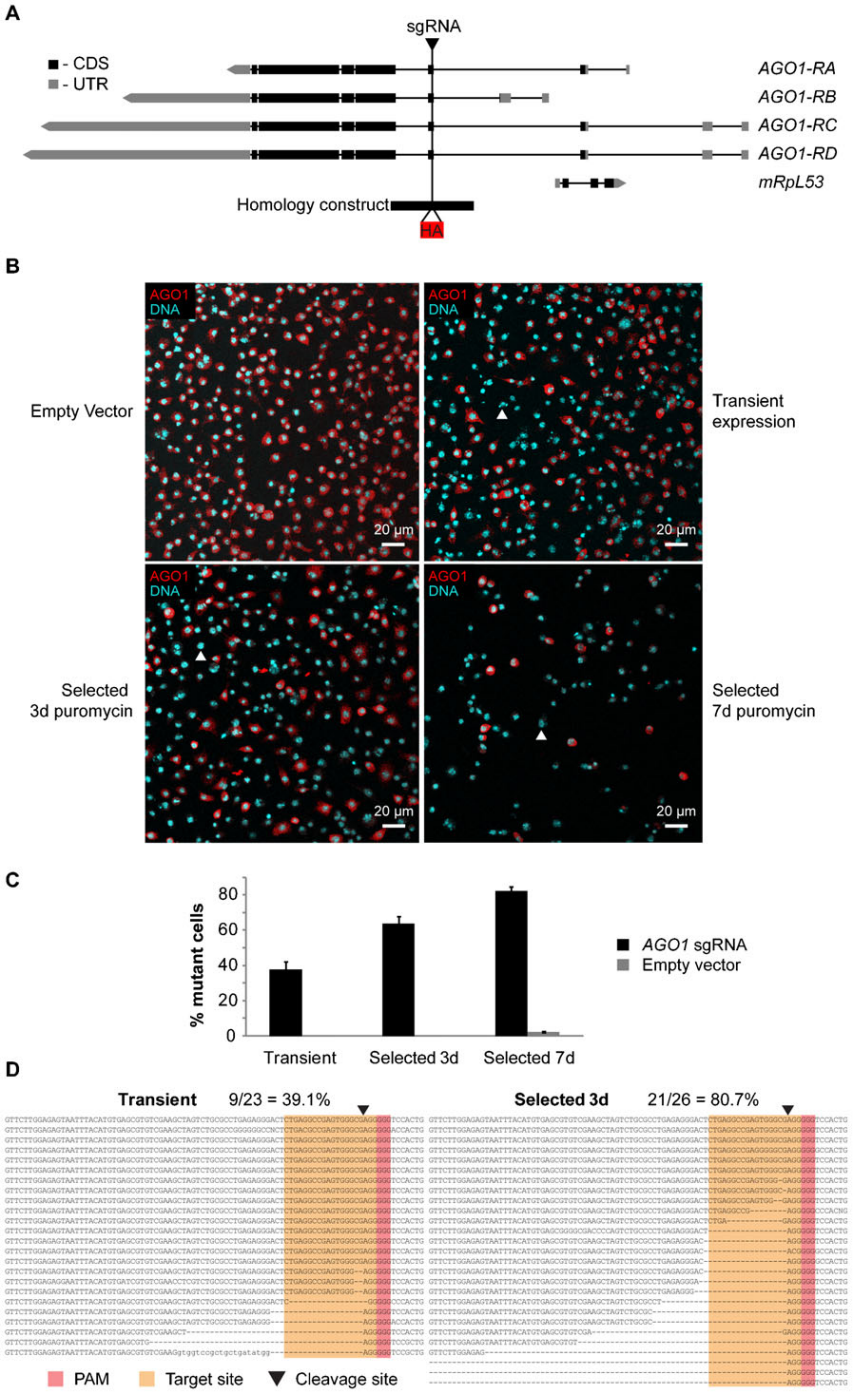
Mutagenesis of the *AGO1* gene. (A) Schematic of the *AGO1* locus. Exons are indicated by boxes, coding sequence in black (CDS) and untranslated regions in grey (UTR). Different splice isoforms and an overlapping gene are indicated on different lines (*AGO1*-*RA*, *RB*, *RC*, *RD*, *mRpL53*). The position of the sgRNA target site is indicated by a black triangle, and the homology construct by a black rectangle, showing the inserted HA tag in red. Primer sequences are indicated in supplementary material Table S1. (B) Efficient mutagenesis of the *AGO1* gene. Images show immunostaining with an anti-AGO1 antibody (red) counterstained for DNA to highlight nuclei of all cells (cyan). The upper left panel indicates cells transfected with Cas9 but lacking sgRNA (empty vector) analysed after selection for 7 days in puromycin. Other panels show cells transfected with the *AGO1* sgRNA and analysed 3 days post transfection (upper right, transient expression), or selected for a further 3 or 7 days in puromycin (bottom panels, selected 3 d or 7 d puromycin). Cells are clearly visible that lack staining for AGO1 protein, but maintain nuclear staining in those cells transfected with the *AGO1* sgRNA (examples indicated with white arrowheads). See also supplementary material Fig. S1. (C) Quantification of mutagenesis efficiency at the *AGO1* gene. Cells that lacked visible staining for AGO1 protein were counted upon transfection with vector expressing *AGO1* sgRNA (black), or lacking sgRNA expression (grey). Cells were analysed 3 days after transfection (transient) or after a further 3 or 7 days selection in puromycin (Selected 3 d or 7 d). Values were expressed as % mutant cells, and error bars indicate 95% confidence intervals of at least 4 biological repeats of at least 200 cells per repeat. (D) Sequencing of indel mutations within *AGO1*. PCR products spanning the cleavage site (black triangle) were cloned and sequenced from cells 3 d after transfection with 2 µg *y*1 sgRNA vector (Transient), or after a further 3 d selection in puromycin (Selected 3 d). Deletions in 9/23 (39.1%, Transient) or 21/26 (80.7%, Selected 3 d) clones were observed at the expected site. Target site is highlighted in orange, and PAM in red. The first line of each alignment indicates the wt sequence. Scale bars: 20 µm.

A useful application of this technique would be to generate genetic mutations in *Drosophila* cell lines, which hitherto has proved impossible. We attempted to construct null mutations of the *AGO1* gene using a sgRNA targeted to the first exon that is common to all transcript variants (Fig. 4A). The target site was chosen to be close to the beginning of the open reading frame in order that frameshift mutations produced would result in a non-functional protein.

Mutagenesis was monitored by immunostaining of cell populations with an anti-AGO1 antibody, which showed that upon transient transfection up to around 35% of cells contained homozygous null mutations in the *AGO1* gene after 3 days. As expected, this proportion increased substantially upon selection, reaching a maximum of 82% mutant cells after selection for a further 7 days in puromycin (Fig. 4B,C; supplementary material Fig. S1). These results were further confirmed by sequence analysis of PCR products spanning the sgRNA target site, which showed that after transient expression, 39.1% of alleles had indel mutations at the expected cleavage site, which increased to 80.7% after selection in puromycin, consistent with the results from immunostaining (Fig. 4D). This suggests that selection for Cas9 expression with puromycin will allow the mutation of protein-coding genes at high efficiency. Further selection resulted in a loss of *AGO1* mutant cells, and after 12 days of selection no mutant cells were visible. This is likely due to a detrimental effect of *AGO1* mutation on cell viability, resulting in wild-type cells outcompeting mutant cells within a population, since similar effects were not observed when Cas9 was expressed alone, or using a sgRNA targeting the *yellow* gene. It is also consistent with previous studies of RNAi-mediated knockdown of AGO1 levels, which show a highly reduced growth rate (Rehwinkel et al., 2006).

### Tagging of the *AGO1* gene

**Fig. 5.**
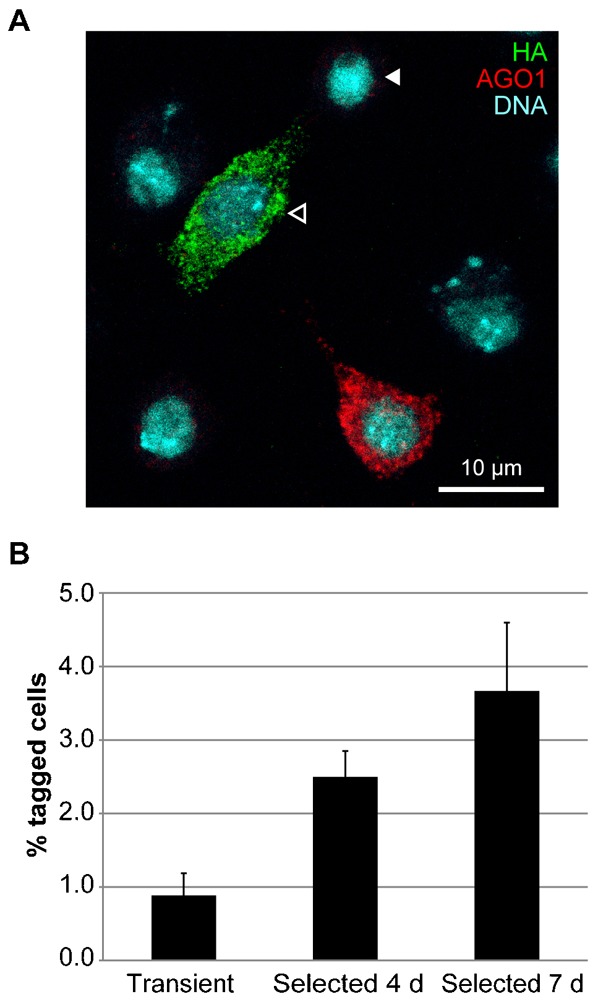
Epitope tagging of the *AGO1* gene. (A) Tagging of AGO1 with a HA epitope tag. Cells transfected with the *AGO1* sgRNA and homology construct shown in Fig. 4A were analysed by immunostaining with anti-AGO1 (red) and anti-HA (green) antibodies and counterstained for DNA (cyan). Staining with anti-HA antibody is clearly observed in some cells (open arrowhead), with a cytoplasmic distribution similar to that of AGO1. Many cells show no AGO1 staining (closed arrowhead) due to homozygous mutation of the endogenous *AGO1* gene. (B) Quantification of AGO1 tagging efficiency. Number of cells staining with anti-HA antibody were quantified 3 days after transfection (transient), or after a further 4 or 7 days selection in puromycin (Selected 4 d or 7 d). Values were expressed as a percentage of the total number of cells (*AGO1* wt and mutant), and error bars indicate 95% confidence intervals of at least 4 biological repeats of at least 200 cells per repeat. Scale bar: 10 µm.

We further investigated whether homologous recombination could be used to insert an hemagglutinin (HA) epitope tag into the AGO1 protein, which could then be used to detect or purify endogenous AGO1 protein in the cell. Approximately 1 kb homology arms were designed to insert the HA sequence in frame with the *AGO1* coding sequence, and our targeting construct was co-transfected with the *AGO1* sgRNA/Cas9 vector. After transient transfection, around 1% of cells were observed to stain with an anti-HA antibody (Fig. 5A). This proportion increased upon selection in puromycin to a peak of approximately 4% (Fig. 5B). The speckled cytoplasmic staining pattern observed with the anti-HA antibody was similar to that observed with the anti-AGO1 antibody, although we did not observe staining of tagged cells with the anti-AGO1 antibody. This is likely due to epitope masking or deletion upon insertion of the tag. However, sequencing across the integration sites confirmed the expected homologous integration and tagging event.

## Discussion

We have described an efficient system for generating genetic mutations in S2 cells that involves simple and rapid cloning of a pair of annealed 23–24 nt oligonucleotides, followed by transfection and selection to enrich for mutant populations. Efficiencies of up to 80% homozygous mutations in protein coding genes could be achieved. This permits the analysis of genetic mutations in *Drosophila* cell lines for the first time. The approach can be used to assess the functionality of different sgRNAs before their use *in vivo*. Indeed, efficiencies of cleavage by different sgRNAs were found to differ substantially. This may be due to many reasons, including chromatin context of the target site, secondary structures within the sgRNA or the stability of sgRNA base-pairing with the DNA. Further studies with large numbers of sgRNA sequences will be necessary to define the rules of sgRNA design needed for efficient targeting.

Additionally we demonstrate the feasibility of homologous integration using relatively short 1 kb homology arms that are easy to generate and whose integrations are straightforward to detect by PCR. This approach could also be used to insert fluorescent or epitope tags into the endogenous copy of any gene, to enable affinity purification or detection of endogenous protein. We observe an efficiency of 1–4%, which is nevertheless sufficient to generate clonal populations either by serial dilution of cells (Nilsen and Castellino, 1999), or by using fluorescent activated cell sorting (FACS) to enrich for integration events such as GFP tagging of endogenous genes. It would also be possible to facilitate selection of homologous integrants by the incorporation of positive and negative selectable markers within and outside the homology arms in the targeting vector, as has been used in other systems (Huang et al., 2008; Mansour et al., 1988).

Upon selection in puromycin, it is probable that the non-integrated allele will be mutated due to indels created by imperfect NHEJ, hence the tagged protein will remain the only functional copy within the cell, which also enables the functionality of the tagged protein to be tested.

The application of the CRISPR/Cas9 system to *Drosophila* cell lines now also permits the construction of targeted deletions (Gratz et al., 2013; Kondo and Ueda, 2013; Ren et al., 2013) or insertions of recombinase sites to allow site-specific integrations (Groth et al., 2004). It also enables use of the various adaptations of the CRISPR/Cas9 system, which have been used to either activate (Maeder et al., 2013; Perez-Pinera et al., 2013) or repress transcription (Qi et al., 2013), to modify chromatin, or to recruit other protein or RNA molecules to specified DNA sequences.

The ease, speed and cost of generating sgRNA expression constructs should enable the production of genome-wide libraries of vectors that target the complete set of protein-coding genes using oligonucleotide printing technologies. If it is possible to screen or select for cellular phenotypes, for example using FACS or drug resistance gene expression, this should allow genome-wide screening of genetic mutants in S2 cells. The efficiency of mutagenesis should be sufficiently high to perform population-based phenotype measurements in a 96 or 384 well format, and to be complementary to currently available RNAi systems. This newfound ability to create stable genetic variants in *Drosophila* cell lines should now provide an adaptable and powerful system to study and screen for gene function at the cellular level.

## Materials and Methods

### Vector construction

A *Drosophila* U6 promoter followed by *Bsp* QI cloning sites and the remainder of the sgRNA backbone was generated as a GBlock (Integrated DNA Technologies), and cloned into the *Bgl* II site of the pAc-STABLE1-Puro vector (González et al., 2011). The human codon-optimised Cas9 was PCR amplified with Phusion DNA polymerase (New England Biolabs (NEB)) from pX330 (Mali et al., 2013b) and cloned as an *Eco* RI/*Hind* III fragment into the pAc-sgRNA-STABLE1-Puro vector. Oligonucleotide sequences are indicated in supplementary material Table S1.

### sgRNA production and ligation

sgRNA target sequences were selected as 20 nt sequences preceding an NGG PAM sequence in the genome. Overhangs were added to allow ligation into the vector. Two oligonucleotides for each target were synthesised and annealed by mixing 10 µl each oligonucleotide (100 µM) and 20 µl 2× annealing buffer (20 mM Tris, 2 mM EDTA, 100 mM NaCl, pH 8.0), and slowly cooling from 98°C to room temperature over a period of approximately 2 h. 1 µl of this mixture was phosphorylated in a 10 µl reaction with 1 µl T4 polynucleotide kinase (New England Biolabs), in T4 DNA ligase buffer at 37°C for 30 min and then diluted 10× in water. 2 µg pAc-sgRNA-Cas9 vector was digested with 20 U *Bsp* QI (NEB) for 2 h at 50°C, and treated with 10 U calf intestinal alkaline phosphatase for 10 min at 37°C (NEB) before PCR purification (Qiagen). Ligations were performed with approximately 50 ng vector and 2 µl annealed diluted oligonucleotides with T4 DNA ligase (NEB) in a 10 µl reaction volume for 2 h at 18°C, and transformed into chemically competent *E. coli* DH5α cells (Life Technologies). Positive clones were selected by colony PCR with a common U6F and specific sgR primers, and sequenced with U6F (supplementary material Table S1).

### S2 cell culture and transfection

S2R+ cells (Drosophila Genomics Resource Center) were grown in Schneider's medium (Sigma) supplemented with 10% FBS (Life Technologies) at 25°C. For transfection, cells were plated at 2×10^6^ cells per well of a 6-well dish, and a total of 2 µg DNA was transfected into each well using Fugene HD (Promega) at a 1:3 ratio (µg:µl), following manufacturer's instructions. For experiments with targeting constructs, 1 µg of expression vector and 1 µg targeting vector or homology oligonucleotide were used. Transfections were analysed after 3 days, and selection was performed subsequently in 5 µg/ml puromycin (Sigma). RNA extractions were performed using a miRNeasy kit (Qiagen).

### Estimation of homologous targeting efficiency

DNA extractions were performed by incubation in lysis buffer (10 mM Tris-HCl pH 7.5, 10 mM EDTA, 10 mM NaCl, 0.5% N-lauroylsarcosine) with 200 µg/ml proteinase K at 55°C for 2 h, followed by phenol:chloroform extraction and ethanol precipitation. PCRs across the homology arms and outside the integration site were performed with 100 ng DNA in 20 µl reactions and cycle numbers adjusted so that amplification was within the linear range. A standard curve consisting of a 2 fold serial dilution of each purified product was used to quantify amounts of each product, after quantification of gel images with Image J. Integration efficiency was calculated by comparison with PCR products outside of the homology arms. Sequencing of PCR products was performed after treatment with ExoSAP-IT (Affymetrix). Oligonucleotide sequences are shown in supplementary material Table S1.

### High resolution melt analysis

Oligonucleotides were designed to give 100–200 nt products spanning the presumed CRISPR cleavage site using Vector NTI (Invitrogen) (supplementary material Table S1). PCR was performed with Hotshot Diamond PCR mastermix (Clent Lifescience) in 10 µl reactions with 1 µl gDNA, 5 µl Hotshot diamond mastermix, 200 nM each oligonucleotide and 1 µl LC Green Plus dye (Idaho Technology). Reactions were cycled on a GStorm thermal cycler (95°C 5 min, 45 cycles of {95°C 20 s, 60°C 30 s, 72°C 30 s}, 95°C 30 s, 25°C 30 s, 10°C ∞). Thermal melt profiles were collected on a LightScanner (Idaho Technology) (70–98°C, hold 67°C) and analysed with the LightScanner Call-IT software.

### Immunofluorescence

Approximately 1×10^6^ cells were transferred to 24 well plates containing circular coverslips, and left to settle for a minimum of 2 h. Cells were washed twice in 0.5 ml PBS, fixed in 0.5 ml 4% paraformaldehyde (Sigma) in PBS for 10 min and washed a further two times in 0.5 ml PBT (PBS + 0.1% Triton X-100). Blocking was performed in PBTS (PBT + 5% normal horse serum) for 1 h, and primary antibodies added in PBTS overnight at 4°C (anti-AGO1 (Siomi, 1B8) 1:1000, anti-HA (Roche, 3F10) 1:1000). Cells were washed 2×5 min in PBT, 1×10 min in PBTS and secondary antibodies added (α-mouse Cy5 1:1000, α-rat Alexa 488 1:1000) for 4 h at room temperature in PBTS. Nuclei were stained with 10 µg/ml Hoescht 33342 for 10 min in PBT, and cells washed twice for 10 min in 0.5 ml PBT before mounting in Dako fluorescent mounting medium. Images were collected on a Leica SP5 confocal microscope, and images processed with LAS-AF software (Leica).

## Supplementary Material

Supplementary Material
