# Supplementary material for: Mutagenesis and homologous recombination in Drosophila cell lines using CRISPR/Cas9
Source: Biol Open. 2013 Dec 6;3(1):42–9. doi: 10.1242/bio.20137120 (PMC3892159; doi:10.1242/bio.20137120)
Supplement: Supplementary Material [file supp_3_1_42__index.html]

Mutagenesis and homologous recombination in Drosophila cell lines using CRISPR/Cas9 — Mutagenesis and homologous recombination in Drosophila cell lines using CRISPR/Cas9 — Supplementary Material 

# Mutagenesis and homologous recombination in *Drosophila* cell lines using CRISPR/Cas9

## bio.20137120 Supplementary Material

**Files in this Data Supplement:**

- Supplementary Material - Andrew R. Bassett et al. doi: 10.1242/bio.20137120
