# Supplementary material for: Mutagenesis and homologous recombination in Drosophila cell lines using CRISPR/Cas9
Source: Biol Open. 2013 Dec 6;3(1):42–9. doi: 10.1242/bio.20137120 (PMC3892159; doi:10.1242/bio.20137120)
Supplement: Supplementary Material [file supp_bio.20137120_bio.20137120-s1.pdf]

Biology Open

Supplementary Material

Andrew R. Bassett et al. doi: 10.1242/bio.20137120

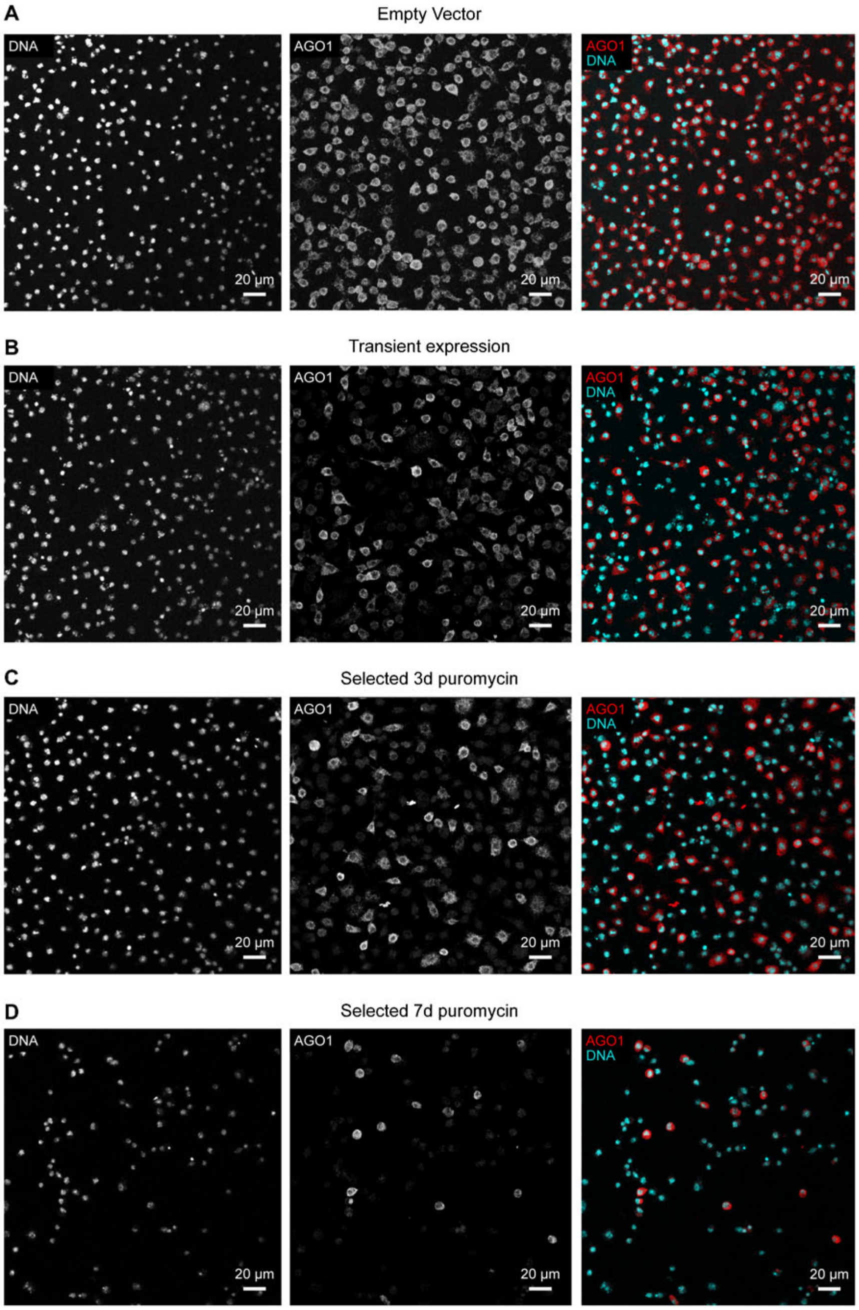

Fig. S1. Individual channels of *AGO1* mutagenesis. The individual channels of DNA staining (left panels) and anti-AGO1 staining (middle panels) are shown alongside the merged images (right panels) as shown in Fig. 4B. (A) Cells transfected with Cas9 but lacking sgRNA (empty vector) analysed after selection for 7 days in puromycin. (B) Cells transfected with the *y*1 sgRNA and analysed 3 days post transfection (transient expression). (C) Cells selected for a further 3 days in puromycin (selected 3 d puromycin). (D) Cells selected for 7 days in puromycin (selected 7 d puromycin). Cells are clearly visible that lack staining for AGO1 protein. Scale bars: 20 µm.

Table S1. Oligonucleotides used in this study.

Primer name	Sequence (5′–3′)
**Expression vector construction**	
U6sgRNA	gttcgacttgcagcctgaaatacggcacgagtaggaaaagccgagtcaaatgccgaatgcagagtctcattacagcacaatcaactcaagaaaaactcgacactttttttaccatttgcacttaaatcctttttttattcgttatgtatactttttttggtccctaaccaaaacaaaaccaaactctcttagtcgtgcctctatatttaaaactatcaatttattatagtcaataaatcgaactgtgttttcaacaaacgaacaataggacactttgattctaaaggaaattttgaaaatcttaagcagagggttcttaagaccatttgccaattcttataattctcaactgctctttcctgatgttgatcatttatataggtatgttttcctcaatacttcggaagagcgagctcttccgttttagagctagaaatagcaagttaaaataaggctagtccgttatcaacttgaaaaagtggcaccgagtcggtgcttttt
U6F	TTagatctGTTCGACTTGCAGCCTGAAATACG
sgRNAR	TTagatctAAAAAAGCACCGACTCGGTGC
Cas9F	TTgaattcATGGACTATAAGGACCACGACGGA
Cas9R	TTTaagcttgcctgatccCTTTTTCTTTTTTGCCTGGCCGG
**sgRNAs**	
y1sgF	TTCGgttttggacactggaaccg
y1sgR	AACcggttccagtgtccaaaacC
AGOsgF	TTCGctgaggccgagtgggcgagg
AGOsgR	AACcctcgcccactcggcctcagC
**HRMA**	
yHMA_F	ATACAGCTGGAGATTGCGCCA
yHMA_R	CCAGGTAGCTCGTATCTCCGAATT
CG14073_F	AGTCCGGCGCCCACACCTGT
CG14073_R	GGGCGCATTTGTTGCACCGT
**Homology arm generation**	
yL_195F	TTactagtGTTTGGCCCTGCTAATTCTCCT
yL_443F	TTactagtCACGGCAGTTACCATTGCTTATG
yL_1002F	TTactagtCGAGGTTTTAGGACTGAAAGAGCAC
yL_1813F	TTactagtGCCCAGCCTTGAGGTCTCTTT
yL_R	tttGGCCGGCCACAGCCGACCACACTCATCCA
yR_F	TTagatctGGGTTTTGGACACTGGAACCG
yR_230R	TTggcgcgccAGCAATCAAGCCGTATCCCAA
yR_464R	TTggcgcgccGGTTTCATCCCTCAAAATCCTCG
yR_888R	TTggcgcgccCCATTGGCAAAACGGCTTG
yR_1453R	TTggcgcgccCCGCTTTTTCCGCTCAAGA
AgoUpF	actctggccggcgtagtcgggcacgtcgtaggggtaTGACTCTATAAAAAGAAAAGGTAATC
AgoUpR	GGCCTGCGTCTCGAATTTATTC
AgoDownF	CTCGTCCGAGATTCGGGCG
AgoDownR	tacccctacgacgtgcccgactacgccggccagagtCAGTGGACCCCCTCGCCC
**To validate homologous integration**	
yL_checkF	CCGAAACACCCAAACTTCTGAC
yR_checkR (aR)	GCGAAACCCATCCATATGACAG
InsOut3 (aF)	AGGGTTAATTGCGTCACTTAGTCAC
InsOut5	TCTCTAATTGAATTAGATCCCCGGG
yContF (bF)	CGCACAACGGTAGACGAAGC
yContR (bR)	TTGCCGATGCACCGAAAA
AgoCheckF	TGATTGGCGCGCAGCACAA
AgoCheckR	ACGGGGGTTAATCTCTGGCGTC
HAF	TACCCCTACGACGTGCCCGACTACGCC
HAR	GGCGTAGTCGGGCACGTCGTAGGGGTA

Biology Open
